# MiR-335 Regulates *Hif-1α* to Reduce Cell Death in Both Mouse Cell Line and Rat Ischemic Models

**DOI:** 10.1371/journal.pone.0128432

**Published:** 2015-06-01

**Authors:** Fu Jia Liu, Prameet Kaur, Dwi S. Karolina, Sugunavathi Sepramaniam, Arunmozhiarasi Armugam, Peter T. H. Wong, Kandiah Jeyaseelan

**Affiliations:** 1 Department of Biochemistry, Yong Loo Lin School of Medicine, National University Health System, National University of Singapore, 8 Medical Drive, 117597, Singapore, Singapore; 2 Department of Pharmacology, Yong Loo Lin School of Medicine, National University Health System, National University of Singapore, 10 Medical Drive, 117597, Singapore, Singapore; 3 Department of Anatomy and Developmental Biology, School of Biomedical Sciences, Faculty of Medicine, Nursing and Health Sciences, Monash University, Clayton, Victoria, 3800, Australia; Indian Institute of Integrative Medicine, INDIA

## Abstract

Hypoxia inducible factor-1α facilitates cellular adaptation to hypoxic conditions. Hence its tight regulation is crucial in hypoxia related diseases such as cerebral ischemia. Changes in hypoxia inducible factor-1α expression upon cerebral ischemia influence the expression of its downstream genes which eventually determines the extent of cellular damage. MicroRNAs are endogenous regulators of gene expression that have rapidly emerged as promising therapeutic targets in several diseases. In this study, we have identified miR-335 as a direct regulator of hypoxia inducible factor-1α and as a potential therapeutic target in cerebral ischemia. MiR-335 and hypoxia inducible factor-1α mRNA showed an inverse expression profile, both *in vivo* and *in vitro* ischemic conditions. Given the biphasic nature of hypoxia inducible factor-1α expression during cerebral ischemia, miR-335 mimic was found to reduce infarct volume in the early time (immediately after middle cerebral artery occlusion) of embolic stroke animal models while the miR-335 inhibitor appears to be beneficial at the late time of stroke (24 hrs after middle cerebral artery occlusion). Modulation of hypoxia inducible factor-1α expression by miR-335 also influenced the expression of crucial genes implicated in neurovascular permeability, cell death and maintenance of the blood brain barrier. These concerted effects, resulting in a reduction in infarct volume bring about a beneficial outcome in ischemic stroke.

## Introduction

Stroke is one of the leading causes of death and adult disability worldwide. microRNAs (miRNAs) have been found to be involved in stroke pathogenesis [1] and to date, numerous miRNAs have been identified to participate in the molecular processes involved in the ischemic cascade [2]. However, the interaction of these miRNAs and their specific target mRNAs during cerebral ischemia is poorly understood. So far rt-PA (recombinant tissue plasminogen activator) is the only FDA approved drug used to treat ischemic stroke. Its narrow therapeutic window of 4.5 hrs and associated risks such as hemorrhagic transformation have limited its therapeutic potential to only 8% of the ischemic stroke population [3]. Hence, there is a pressing need to search for an alternative therapy for ischemic stroke.

Hypoxia occurs immediately upon cerebrovascular occlusion and contributes to the progression of ischemic cascade. Hence, hypoxia inducible factor-1α (*HIF-1α*), an essential regulator of hypoxic events could be a useful target for the treatment of ischemic stroke. *HIF-1α* mediates important endogenous adaptive mechanisms in order to maintain oxygen homeostasis and also facilitates cellular adaptation to low oxygen conditions by regulating more than 80 downstream genes [4–6]. These genes code for molecules participating in angiogenesis, erythropoiesis, energy metabolism, apoptosis and neuronal stem/progenitor cells (NSPC) proliferation [7–9]. HIF-1α was found to be an important player in cerebral ischemia [10–13]. It displayed biphasic expression and regulate some of its downstream genes [14,15] upon ischemic stroke. Inhibition of *Hif-1α* in the early phase of ischemic stroke by using siRNAs has been found to bring about reduction of infarct damage [14].

Taguchi *et al* [16] first reported that miR-17-92 cluster directly targets *HIF-1α* in lung cancer cells. Subsequently, several other miRNAs including miR-20b, -22, -138, -155, -199a-5p -429 and -519c were also shown to regulate *HIF-1a* in cancer or hypoxic conditions [17–23]. Interestingly, among these miRNAs, miR-155 [19] and miR-429 [23] were shown to directly target *HIF-1α* mRNA 3’UTR, and found to be involved in *HIF-1α* negative-feedback loop. However, the implication of modulating the expression of *HIF-1α* using miRNAs in cerebral ischemia remains unexplored. In this study, we aim to identify the miRNAs that could directly regulate *Hif-1a* expression and bring about a favorable outcome through the reduction of infarct size in cerebral ischemia.

## Materials and Methods

### Rat model of middle cerebral artery occlusion using an embolus (eMCAo) and quantitation of infarct volume

Male Wistar rats (280g - 320g) were obtained from the Laboratory Animal Centre (National University of Singapore, Singapore) and maintained on an *adlibitum* intake of standard laboratory chow and drinking water under controlled temperature and 12 hrs light/dark cycles. All animals used in this study were handled strictly in accordance to the recommendation of the Council for International Organisation of Medical Sciences on Animal Experimentation (World Health Organisation, Geneva, Switzerland) and the Institutional Animal Care and Use Committee’s (National University of Singapore) guidelines. The animal protocol/procedure was approved by the National University of Singapore’s Institutional Animal Care and Use Committee (IACUC Protocol Number: 081/09). All surgeries were performed under intraperitoneally administered ketamine/xylazine (7.5mg/1mg per 100g) anaesthesia. Analgesic (buprenorphine 0.02mg/kg) and antibiotic (Baytril 6mg/kg) were injected subcutaneously after surgery for three days (once per day) to minimize animal distress/suffering. Animals were randomly grouped into control and eMCAo. Middle cerebral artery occlusion (MCAo) was induced by injecting an embolus into the middle cerebral artery as described by Zhang *et al* [24]. Rats in the eMCAo group were euthanized at time points, 0, 3, 6, 12, 24, 48, 72, 120 and 168 hrs. A minimum number of six animals (n = 6) were used for each group. Animals were sacrificed by euthanasia in a carbon dioxide chamber. Without pre-charging the chamber, the rodents (one at a time) were placed in a transparent chamber and 100% CO_2_ was introduced slowly for about 7mins. The rodents were verified for death by checking for absence of heartbeat, absence of breathing, presence of paleness of mucosal tissues and absence of reflex reaction upon toe pinching. We observe the rats for few more minutes before and the brains were then removed and used for subsequent experiments or stored at -80°C.

The detailed procedure and quantitation of the neurological deficiency were performed as described previously [25].

### Extraction of total RNA

Total RNA (+ miRNA) was extracted from brain tissues by a single-step method using Trizol reagent according to manufacturers’ protocol (Invitrogen, Life Technologies, USA). The concentration and integrity of RNA were determined using Nanodrop ND-1000 spectrophotometry (Nanodrop Tech, Rockland, Del) and denaturing gel electrophoresis.

### MiRNA profiling

miRNA array was performed as described previously [25]. Total RNA (500 ng) was 3′-end-labelled with Hy3 dye using the miRCURY LNA Power Labeling Kit (Exiqon, Denmark). The labelled miRNAs were hybridized for 16–18 hrs, on miRCURY LNA Arrays, using MAUI hybridization system according to manufacturer’s protocol (Exiqon, Denmark). The microarray chips were then washed and scanned using InnoScan700, microarray scanner and analysed using the Mapix Ver4.5 software (Innopsys, Carbonne, France).

### Cloning of *Hif-1α* 3'UTR and dual luciferase reporter assay

The 3'UTR of *Hif-1α* (*Rattus norvegicus* hypoxia inducible factor-1α NM_024359.1) was amplified by PCR using gene specific primers. The PCR products were cloned into Firefly-luc-expressing vector pMIR-REPORT (Ambion, Austin, TX). Plasmid transfection procedure was adapted from Sepramaniam *et al* [26]. HeLa cells were selected for the study as they show relatively high transfection efficiency (>80%) when compared to post-mitotic neurons (30%) [27]. Furthermore, Bruning *et al* [19] have also used the same cell line for a luciferase reporter assay involving miR-155 and *HIF-1α*. In brief, HeLa cells cultured in 24-well plates were transfected with 30 nM anti or mimic microRNA for 3 hrs followed by 200 ng/well pMIR-REPORT constructs for 3 hrs [26]. The cells were left to recover in a CO_2_ incubator for 48 hrs before being lysed for measurement of luciferase activity. Dual luciferase assay (Promega, USA) was used to quantitate the effects of miRNA interaction with the 3' UTR of *Hif-1α*. The assay was performed according to manufacturer’s protocol (Promega, USA). In all experiments, transfection efficiencies were normalized to those of cells co-transfected with the Renilla-luc-expressing vector pRL-CMV (Promega, USA) at 5 ng/well.

### Primary cortical neuronal cultures

Primary cultures of cortical neurons were established from E15 Swiss albino mouse brains according to Hirai *et al* [28] with slight modifications performed by Kaur *et al* [29]. The cortices were dissected from E15 mouse embryos and washed with Hanks’ balanced salt solution (HBSS; Gibco, Invitrogen, USA). The cortical slices were dissociated with 0.05% (w/v) trypsin in HBSS without Ca^2+^/Mg^2+^ (Gibco, Invitrogen, USA) for 30 min at 37°C and neutralized with 1 mg/ml trypsin inhibitor (Sigma, USA). Single cells were obtained by gentle trituration in Neurobasal medium (Gibco, Invitrogen, USA) supplemented with B27 (Invitrogen, USA), L-glutamine and penicillin-streptomycin (Gibco, Invitrogen, USA). The cells were counted by trypan blue exclusion assay and seeded on to poly-D-lysine coated 24 well plates at a density of 120,000 cells/cm^2^. Cultures were maintained at 37°C with 5% CO_2_ in a tissue culture incubator.

### Oxygen-glucose deprivation

Primary neuronal cultures from day 6 were subjected to oxygen-glucose deprivation (OGD) as previously described [25]. Glucose-free Earle’s balanced salt solution (EBSS) was saturated with a mixture gas of 5% CO_2_, 95% N_2_, with O_2_ maintained at 0.1%, in a ProOx *in vitro* chamber (BioSpherix, USA) at 37°C overnight. Day 6 neuronal cultures were washed twice with the same medium and incubated for 2, 4 and 6 hrs in the chamber. OGD was terminated by replacing the glucose-free EBSS with reperfusion medium (Neurobasal medium with L-glutamine and penicillin-streptomycin, without B27 supplement). Control cultures were treated identically, but without exposure to OGD conditions. During reperfusion, the cells were maintained in a regular 5% CO_2_ incubator for 24 hrs. For anti miR-335 and miR-335 mimic treatment, 4 hrs of OGD time point was selected (as described under results section). Neurons cultured in 24-well plates were subjected to 4 hrs OGD followed by transfection with 30 nM anti miR-335 and miR-335 mimic during the reperfusion period. miRNA mimic and anti miRNA negative controls were used as vehicle controls (mirVana miRNA Mimic Negative Control, 4464058 and mirVana miRNA Inhibitor Negative Control, 4464076; Ambion Inc, USA).

### Morphologic assessment of apoptosis and cell viability using Hoechst/Ethidium homodimer III nuclear staining and fluorescence microscopy

Cells subjected to OGD (and/or anti-miR-335/miR-335 mimic) were stained with Hoechst 33342 and Ethidium Homodimer III (EtHD) dye as described in the manufacturer’s protocol (Biotium, USA.). Stained cells were visualized by fluorescence microscopy (DMIRB, Leica Microsystems Inc, Deerfield, IL USA). Images were captured at 40x objectives and cell morphology was determined as follows; (1) Viable cells had blue-stained normal, smooth nuclei; (2) Apoptotic cells had blue-stained nuclei with fragmented/condensed chromatin. A minimum of 3 fields of at least 100 cells per field were counted to determine the percentage of apoptotic cells from the total number of cells. Experiments were performed in triplicates (n = 3) and carried out at least twice.

### Reverse transcription and real-time quantitative PCR

Reverse transcription followed by real-time quantitative PCR (qRT-PCR) were carried out according to Jeyaseelan *et al* [1]**.** Quantitation of *Hif-1α*, angiopoietin2 (*Angpt2*), BCL2/adenovirus E1B 19kDa interacting protein 3 (*Bnip3*), matrix metalloproteinase-9 (*Mmp9*), plasminogen activator inhibitor 1 (*Pai1*) and vascular endothelial growth factor a (*Vegfa*) mRNAs was performed using SYBR green assay. Specific primer sequences were generated using PrimerExpress Software (Applied Biosystems, USA). For miRNA detection, reverse transcription followed by stem-loop qRT-PCR reactions were performed according to manufacturer’s protocols using miRNA specific stem-loop primer probes (Ambion Inc, USA). Ribosomal RNA (18S rRNA) was used as the endogenous control for the quantitative PCR (qPCR) assays for both mRNAs and miRNAs as it is known to be stable in cerebral ischemic conditions [1,26]. Moreover 18S rRNA has also been previously used as an endogenous control by Bruning *et al* [19] in their study on miR-155 interaction with *Hif-1α*.

### SDS-PAGE and western blot analysis

Forty (40) μg of total protein was resolved using 12% Tris-Tricine SDS-PAGE and Western blot was carried out as described by Sepramaniam *et al* [26]. Membranes were probed with primary antibody (rabbit anti HIF-1α, Abcam, UK) at a concentration of 1: 5,000 in 0.5% blocking solution. β-actin was used as a loading control [26,30].

Secondary antibody (horseradish peroxidase-conjugated goat anti-rabbit, Bio-Rad Laboratories Inc, PA, USA) was used at a dilution of 1:10,000 in 0.5% blocking solution. The membranes were washed and visualized using the enhanced chemiluminescence reagents (SuperSignal West; Thermo Scientific, USA) with variable exposures on Kodak-MS film. Films of Western blots were scanned (Acer SWZ3300U) and intensities of the corresponding bands were quantitated using Image J software (National Institutes of Health, USA).

### *In vivo* injection

*In vivo* administration of miRNAs was done as described previously by Sepramaniam *et al* [26]. Rats were anesthetized and placed in a stereotaxic apparatus (David Kopf Instrument, Tujunga, USA or TSE, Bad Homburg, Germany) with the coordinates of 0.8 mm posterior to the bregma, 1.5 mm lateral to the midline, and 4.5 mm ventral to the surface of the skull for intracerebroventricular (ICV) injection into the left lateral ventricle. Five (5) μl miRNA mimic or anti miRNA (Ambion Inc, USA) were diluted with the equal volume of transfection reagent siPORT NeoFX (Ambion Inc, USA) and the mixture was then incubated 15 min at room temperature (25°C). The RNA/transfection reagent complex was then administered *via* ICV injection using a Hamilton microsyringe into the rat brain. miRNA mimic and anti miRNA (as for OGD studies) were used as vehicle controls.

### Statistical and bioinformatics analyses

Microarray analyses involved multiple sample analysis including background subtraction, *t*-Test/One-way ANOVA analysis and hierarchical clustering [25]. Normalization was performed using the endogenous control, 5S rRNA. Statistical evaluations were performed a) using two-tailed *t*-tests whenever only two sets of data were compared and b) by One-way ANOVA with significance level *p value < 0*.*05* when multiple comparisons were involved. The clustering using hierarchical method was performed with average linkage and Euclidean distance metric. The clustering was generated using TIGR MeV (Multiple Experimental Viewer) software and statistical analysis was performed using Partek Genomics Suite 6.6 (Partek Inc, USA).

To determine the miRNAs that could target *Hif-1a*, a bioinformatics analysis was performed using the online databases such as TargetScan (www.targetscan.org, Release 5.2, June 2011), MiRanda (www.microRNA.org, August 2010 release), Pictar (pictar.mdc-berlin.de/, March 2007 release), Diana microT (diana.cslab.ece.ntua.gr/microT/, 2011 release), miRGen (www.diana.pcbi.upenn.edu/miRGen.html, January 1, 2007(v3) release) and RegRNA (regrna.mbc.nctu.edu.tw/html/prediction.html,Release 1.0) databases [31–34]. miRNAs that were conserved in both rodents and human and predicted by at least two databases were selected for further analyses.

## Results

### *HIF-1α* expression in the eMCAo model

**Fig 1:**
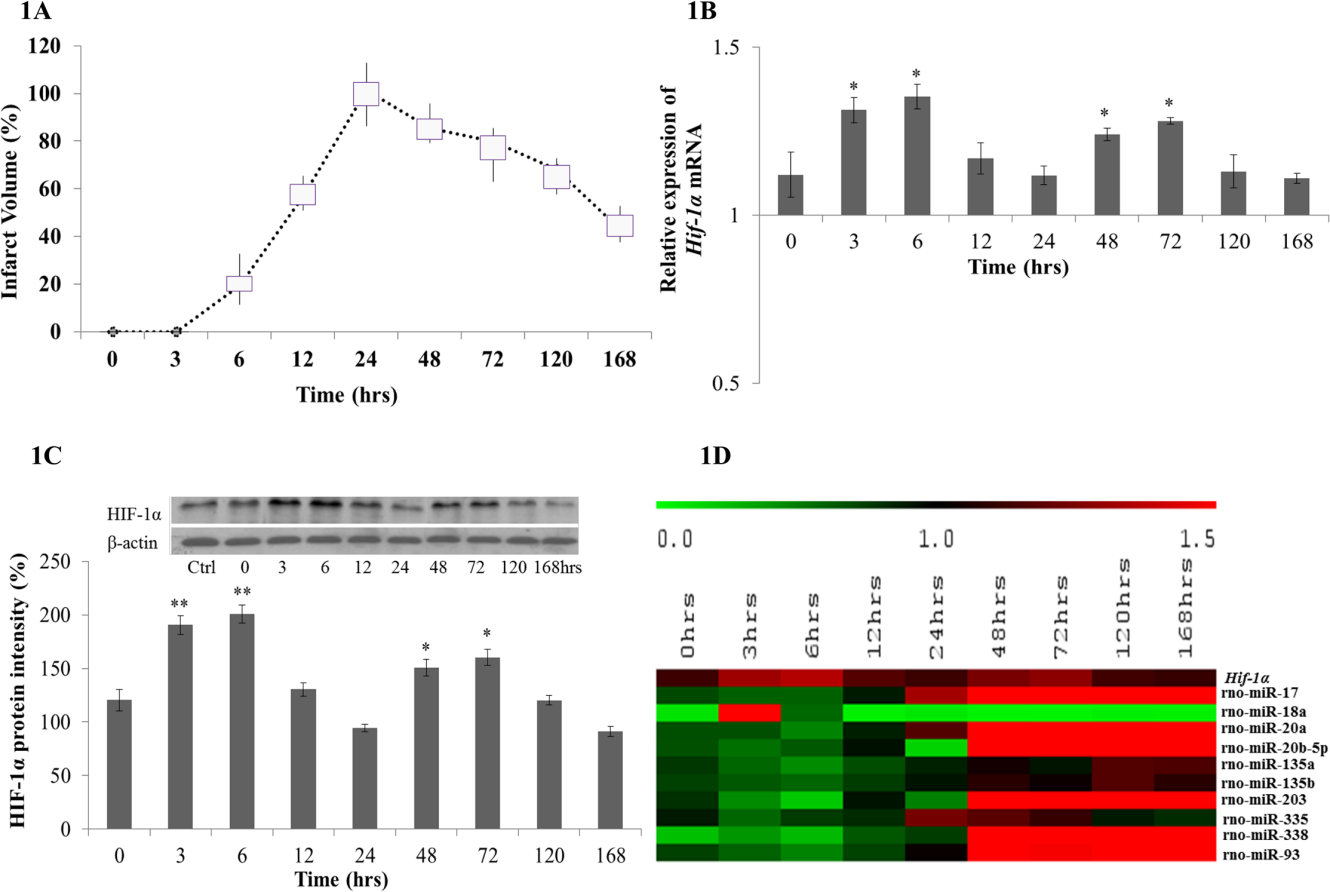
Infarct volume and the expression of HIF-1α mRNA, protein and miRNAs predicted to target Hif-1α at 0, 3, 6, 12, 24, 48, 72, 120 and 168 hrs after eMCAo. (A) Box plot on percentage of infarct volume at each time point after eMCAo. Brain slices were stained with TTC prior to the measurement of the infarct size. Infarct volume at 24 hrs post occlusion reached the maximum (350 ± 30mm^3^) than that of other time points (*p value* < 0.05), thus the infarct volume at 24 hrs was set as 100%. (B) *Hif-1α* mRNA expression at each time point upon cerebral ischemia. *Hif-1α* mRNA was measured by quantitative RT-PCR and presented as relative expression (mean ± SD with n = 3). (C) HIF-1α protein expression at each time point upon cerebral ischemia. Expression was measured by Western blot and the intensities of the bands were quantified using Image J software and presented as percentage of control (%). (D) Heat map of predicted miRNAs and *Hif-1α* expression in eMCAo model. miRNA presented as relative expression of miRNAs from the array data. The differentially expressed miRNAs that were statistically significant (one-way ANOVA *p value* < 0.05) are included. *Hif-1α* mRNA expression presented as relative expression measured by realtime PCR (mean ± SD with n = 3).

Rats subjected to eMCAo (n = 6) were sacrificed randomly at the end of 0, 3, 6, 12, 24, 48, 72, 120 and 168 hrs. Brain slices of these animals were stained with TTC and the infarct volumes on the ipsilateral region were measured. The infarct volume showed a similar pattern as we had reported previously [25], where it peaked at 24 hrs and then declined progressively from 48 hrs until 168 hrs post-occlusion (Fig 1A). In order to observe the expression patterns of *Hif-1α* mRNA and its corresponding protein throughout these time points, the mRNA and protein levels in the ischemic brain tissues were measured. Both mRNA and protein demonstrated a biphasic expression pattern. The *Hif-1α* gene expression increased from the onset of ischemic stroke and peaked at 6 hrs post occlusion and progressively decreased until 24 hrs (Fig 1B). A second peak was observed at 72 hrs followed by a decrease thereafter towards 168 hrs. Similar observations were also noticeable in the protein expression, suggesting a biphasic trend in its expression pattern upon cerebral ischemia (Fig 1C).

### Identification of miRNAs binding to *Hif-1a* mRNA

Bioinformatics search was performed to identify the miRNAs that could target *Hif-1a*. A total of 67 miRNAs was predicted to target *Hif-1a* in rat. Among them, miRNAs that were conserved in both rodents and human and predicted by at least two databases were shortlisted for further studies. The search yielded 12 miRNAs (miR-17, -18a, -20a, 20b-5p, -93, -135a/b, -138, -199a-5p, -203, -335, -338-3p) as potential regulators (Table 1). Ten of them, miR-17, -18a, -20a, -20b-5p, -135a/b, -203, -335, -338-3p and -93 were found to be differentially and significantly (*p*<0.05) expressed in our miRNA profiling analysis of eMCAo rat brain samples (Fig 1D).

**Table 1:** miRNAs predicted to target 3'UTR of *Hif-1*α.

*In silico* prediction of miRNA: *Hif-1α* interaction	miRNAs
**miRNAs conserved in rodent and human (predicted by at least 2 databases)**	**miR-17** ^[^ ^16^ ^]^, **-18a** ^[^ ^16^ ^]^, **-20a** ^[^ ^16^ ^]^, **-20b-5p** ^[^ ^22^ ^]^, **-135a**, **-135b**, -138, -199a-5p, **-203**, **-335**, **-338-3p**, **-93**

MiRNA:*Hif-1α* target prediction was performed and 12 miRNAs, commonly predicted in at least 2 out of the 6 databases used, were selected. Among these, 10 miRNAs which were observed to be significantly expressed in brain samples of rats subjected to eMCAo (*p value<* 0.05) are shown in bold whereas previously validated miRNAs are underlined.

Among the 10 miRNAs, interaction of miR-17, -18a, -20a and -20b-5p with *HIF-1a* had been reported previously in cancer pathogenesis [16,22] whereas the remaining six miRNAs, miR-135a/b, -203, -335, -338-3p and -93 had not been validated in any disease condition.

### MiR-335 and miR-93 directly target *Hif-1α*

**Fig 2:**
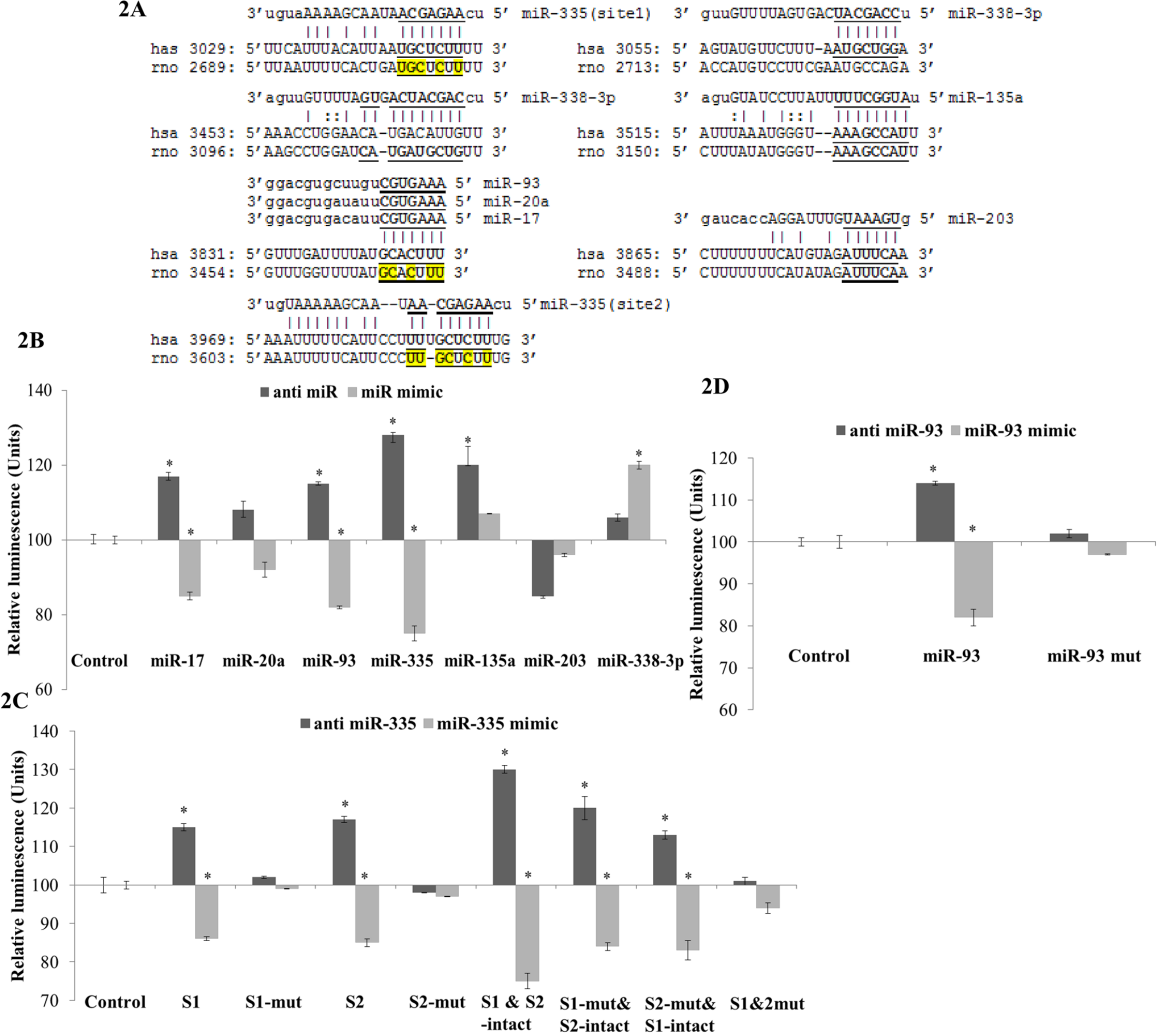
3'UTR sequence of the HIF-1α gene for both rat and human and Dual Luciferase assay for selected miRNAs predicted targeting *Hif-1α*. (A) The sequences were retrieved from NCBI GenBank Database (hsa NM_001530.3 and rno NM_024359.1). The predicted binding site(s) of selected miRNAs (miRNA-17, -20a, -135a, -203, -335, -338-3p and -93) to the 3'UTR of *Hif-1α* is mapped in this figure. The miRNA seed regions are marked in bold and underlined. Nucleotides which were altered for mutational studies are marked in yellow colour. (B) Quantitation of the effects of anti miRNAs and miRNA mimics (including miR-17, -20a, -135a, -203, -335, -338-3p and -93) interaction with the 3' UTR of *Hif-1α*. (C) Quantitation of the effects of anti miR-335 and miR-335 mimic interactions with the normal binding sites and mutated binding sites in 3' UTR of *Hif-1α*. S1 and S2 denoted site 1 and 2 respectively. (D) Quantitation of the effects of anti miR-93 and miR-93 mimic interactions with the normal binding sites and mutated binding sites in 3' UTR of *Hif-1α*. The plasmid constructs together with anti miRNAs and miRNA mimics were co-transfected into HeLa cells. Luminescence for luciferase gene activity in treated samples (anti miRNAs and miRNA mimics) was obtained 48 hrs post-transfection. Relative luminescence was obtained by normalizing the values against control plasmids, pMIR-REPORT without any 3'UTR insert. Data presented as mean ± SD with n = 3. Statistically significant differences are tested at *p value* < 0.05 significance. *p value < 0.05.

In order to establish the direct interaction of the six miRNAs, a luciferase reporter assay was performed. As miR-135a and miR-135b only differs by one nucleotide in a non-seed region, miR-135a was selected for validation studies. miR-17 and -20a were also included in the validation study as positive controls for they were previously reported to be the strongest regulators of *HIF-1α* in cancer pathogenesis [16]. The predicted binding sites of the selected miRNAs are shown in Fig 2A. miR-93 was observed to share the same binding site as miR-17 and miR-20a while miR-335 had two binding sites.

The *Hif-1α* 3’UTR containing the binding sites for these miRNAs were cloned into firefly luciferase reporter plasmids, pMIR-REPORT. These constructs were co-transfected independently with the respective anti and mimic miRNAs into HeLa cells. miR-17 and miR-20a inhibitors and mimics were used as positive controls, since these two miRNAs were previously shown to target *HIF-1α*. Independent transfection of HeLa cells with anti miR-17/-20a/-335/-93 exhibited an increase in the relative luciferase activity, whereas introduction of miR-17/-20a/-335/-93 mimics resulted in a reduction in luciferase activity suggesting that, apart from miR-17 and miR-20a, miR-335 and miR-93 are also direct regulators of *Hif-1α* (Fig 2B). Nevertheless, miR-335 exhibited a stronger interaction when compared to miR-17, miR-93 and miR-20a. Furthermore, interaction between miR-20a and *Hif-1α* was not found to be significant in our study. miR-135a, -203 and -338-3p did not show direct interaction with *Hif-1α* (Fig 2B).

Site-directed mutagenesis was performed to further validate the interaction of the newly established regulators, miR-335 and miR-93 with *Hif-1α* 3’UTR. Loss of the miR-335 and miR-93 recognition sites on the 3’UTR abolished the interactions between the miRNAs and their target (Fig 2C and 2D). These results further confirmed miR-335 and miR-93 as direct regulators of *Hif-1α* and also suggested miR-335 as a stronger modulator than miR-93.

### MiR-335 regulates *Hif-1α* in primary neurons

**Fig 3:**
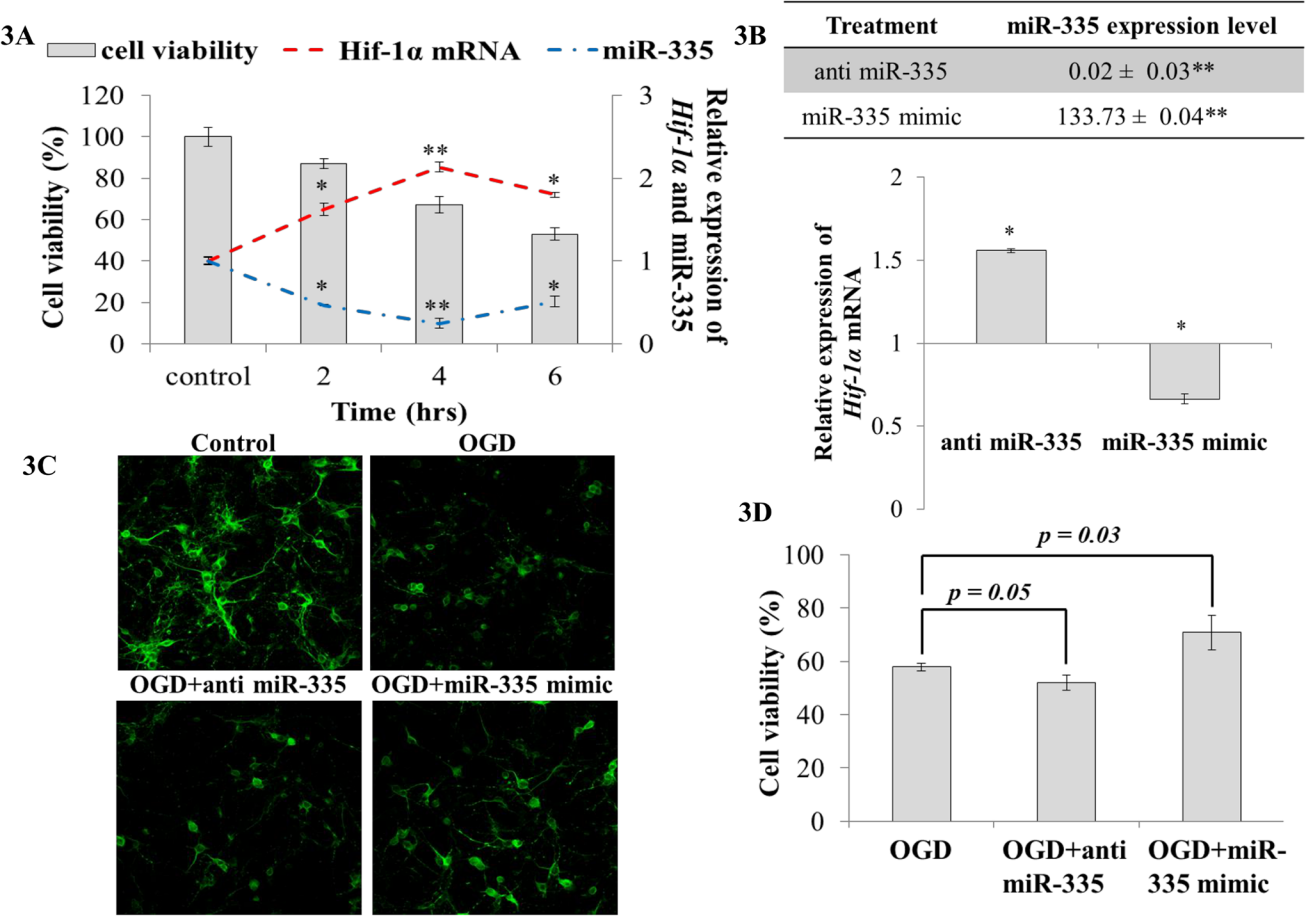
Cell viability and *Hif-1α* expression in primary neuronal culture transfected with anti miR-335 or miR-335 mimic during reperfusion following OGD. **(A)** *Hif-1α* was significantly upregulated upon OGD while miR-335 on the other hand demonstrated a significant downregulation in which both showed maximum changes at 4 hrs OGD. The primary y-axis denotes cell viability whereas the secondary y-axis indicates relative expression of *Hif-1α* and miR-335; **(B)** miR-335 expression significantly increased in the presence of miR-335 mimic (*p* < 0.01) and significantly decreased when cells were transfected with anti miR-335 (*p* < 0.01) during reperfusion. Cells transfected with miR-335 mimic during reperfusion after OGD showed significantly decreased *Hif-1α* expression compared to cells subjected to OGD and transfected with vehicle; **(C)** Neurons subjected to OGD displayed increased apoptosis and degenerated neuritis. Cells transfected with miR-335 mimic after OGD showed increased cell survival and maintenance of neurites as compared to cells subjected to OGD. **(D)** Cells transfected with miR-335 mimic demonstrated significantly reduced cell death compared to cells subjected to OGD and transfected with vehicle (*p* < 0.05). Relative expression (2^-ΔΔCt^) was calculated based on vehicle transfected controls as calibrator and 18S as endogenous control. * *p value* < 0.05; ** *p value* < 0.01.

Having seen that miR-335 as a stronger regulator of *Hif-1α*, we went on to assess its effectiveness in an *in vitro* setting. Primary neuronal cells were subjected to OGD and the changes in *Hif-1α* and miR-335 expression were observed. We found that *Hif-1α* was significantly upregulated upon OGD while miR-335 demonstrated significant downregulation. Maximum changes in expression were noticed at 4 hrs OGD (Fig 3A). Hence, 4 hrs OGD was selected for further studies. During reperfusion following OGD, anti miR-335 and miR-335 mimic were transfected to primary neurons independently and changes in miRNA and *Hif-1α* expression were observed after 24 hrs reperfusion. In primary neurons subjected to OGD, the introduction of anti miR-335 decreased miR-335 level by about 98% (0.02 ± 0.03; Fig 3B) and resulted in an elevation in *Hif-1a* mRNA (Fig 3B). Cell viability and imaging analysis showed that anti miR-335 transfected neurons did not exhibit significant cell death when compared to OGD controls (Fig 3C and 3D). On the other hand, transfection of miR-335 mimic caused a 133.73 fold increase in miR-335 expression (Fig 3B) with a corresponding decrease in the *Hif-1a* mRNA expression (Fig 3B). In addition, a significant reduction in neuronal cell death was noticeable (Fig 3C and 3D).

### MiR-335 mimic reduces infarct volume in acute ischemia

As miR-335 modulation could directly regulate *Hif-1a* and improve cell viability during OGD (*in vitro*), the next attempt was to investigate whether such regulation was reflected in an *in vivo* setting. miR-335 was differentially expressed upon cerebral ischemia in our eMCAo model and it peaked at 24 hrs which corresponded to the maximum infarct volume (Fi 4A). We administered anti miR-335 and miR-335 mimic *in vivo via* ICV injection to ischemic rats immediately following eMCAo.

**Fig 4:**
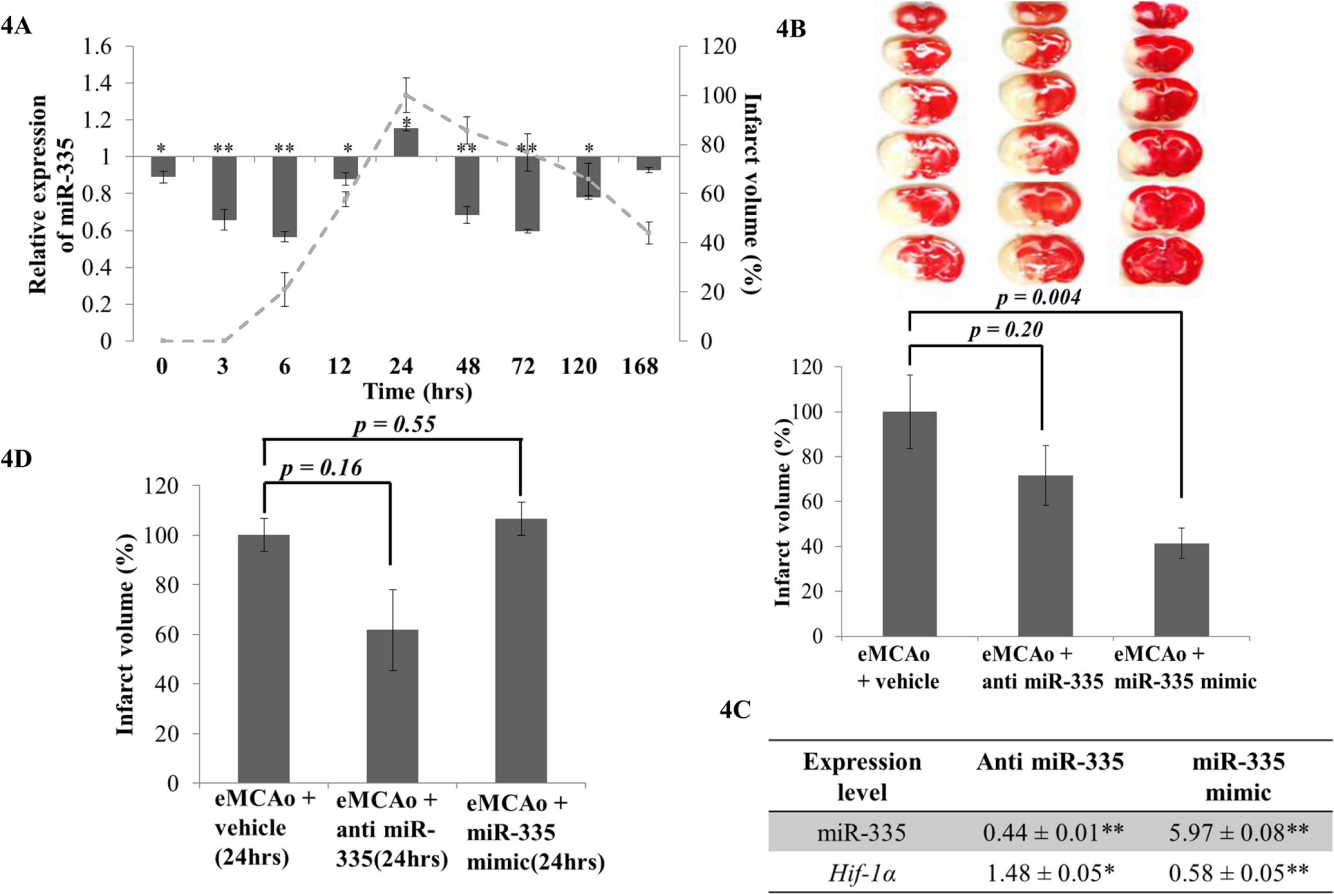
Effects of modulation of expression of miR-335 and *Hif-1α* mRNA in cerebral ischemia. **(A)** miR-335 expression upon cerebral ischemia was measured at 0, 3, 6, 12, 24, 48, 72, 120 and 168 hrs by realtime PCR and relative expression is presented in the graph as a histogram (mean ± SD with n = 3). Infarct volume was plotted as the percentage of infarct volume at 24 hrs (maximum infarct volume) at each time point after eMCAo (—). The primary y-axis denotes relative expression of miR-335 whereas the secondary y-axis signifies the infarct volume; **(B)** TTC was used to stain coronal brain sections (2 mm thick) of rats injected with 50 pmoles of anti miR-335 or miR-335 mimic after eMCAo. Intracerebroventricular (ICV) injections were administered immediately following embolus introduction. Surviving cells were stained in pink while dead cells remained white. Infarct volume of treated rats were expressed as a percentage of the vehicle ± SEM, n = 4. **(C)** *Hif-1α* mRNA expression significantly decreased in miR-335 mimic injection samples (*p value* < 0.01) whereas significantly increased when administration of anti miR-335. **(D)** ICV injections were administered at 24 hrs following embolus introduction. Infarct volume of rats treated with eMCAo + anti miR-335 or miR-335 mimic was expressed as a percentage of the vehicle ± SEM.

Brain samples were harvested at 24 hrs post-occlusion. The mean infarct volumes were measured for each experimental category (Fig 4B). Administration of miR-335 mimic immediately after occlusion reduced the infarct volume by 60%, while administration of anti miR-335 did not result in any significant changes in infarct volume (Fig 4B). The expression level of miR-335 and their target, *Hif-1α*, in the eMCAo rat brain samples were quantitated using real time PCR. Introduction of miR-335 mimics significantly increased their corresponding mature miRNA levels with a reduction in *Hif-1α* mRNA expression (Fig 4C). Nonetheless, the expression of miR-335 reduced when introduction of anti miR-335 in the rat brain samples with a corresponding increase in *Hif-1α* mRNA (Fig 4C).

### Anti-miR-335 reduces infarct volume in the late time of cerebral ischemia

Since we had observed that administration of miR-335 mimic immediately after eMCAo significantly improved infarct volume, we next attempted to determine miR-335’s effect on the late time of cerebral ischemia. To assess this, anti miR-335 and miR-335 mimic were administrated independently *via* ICV injection in eMCAo animals at 24 hrs post-occlusion and brain samples were harvested at 48 hrs (post-occlusion). It was observed that infarct volume was reduced by 40% upon administration of anti miR-335 at 24 hrs post-occlusion (Fig 4D).

### Indirect targets of miR-335 via *Hif-1α*

Having known that *Hif-1α* is a master transcription factor in hypoxic condition, we were then interested in studying the effects on modulating miR-335 expression on the downstream genes in this eMCAo model. We verified the expression of *Angpt2*, *Bnip3*, *Mmp9*, *Pai1* and *Vegfa* genes since they were reported to be regulated by HIF-1α during cerebral ischemia [15,35,36]. It is noteworthy that none of these genes has a binding site for miR-335 at their respective 3'UTRs, yet they are known to be regulated by HIF-1α.

**Fig 5:**
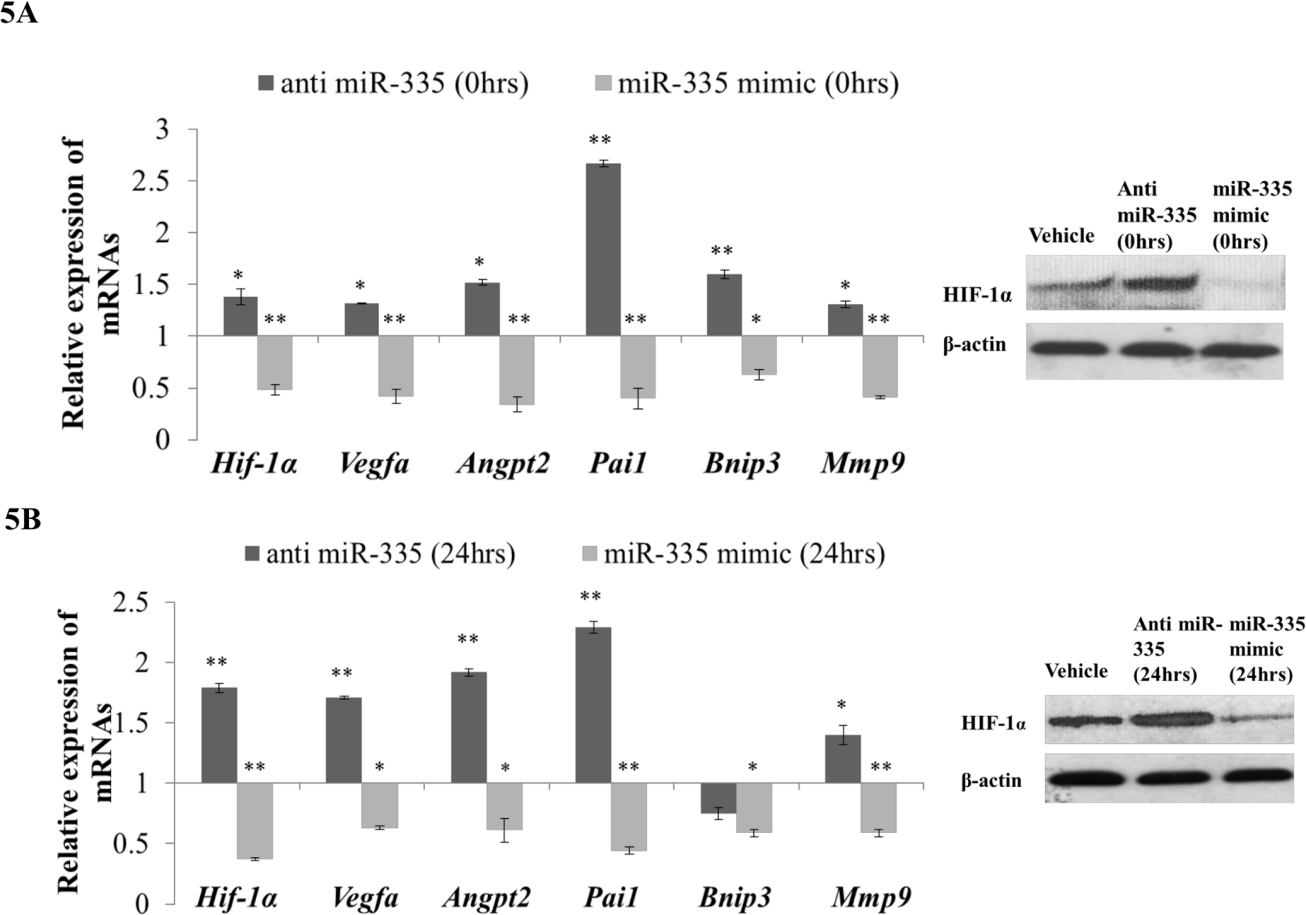
Analyses of *Hif-1α* and its downstream genes expression in eMCAo rats injected with anti miR-335 and miR-335 mimic in the early and late time of cerebral ischemia. (A) *Hif-1α* mRNA expression significantly decreased in miR-335 mimic injection samples (*p value* < 0.01) in early time of cerebral ischemia whereas it significantly increased upon administration of anti miR-335. HIF-1α protein was measured by Western blot and showed a similar expression pattern as *Hif-1α* mRNA. *Angpt2*, *Bnip3*, *Mmp9*, *Pai1* and *Vegfa* mRNA expressions were significantly downregulated in the miR-335 mimic injection samples whereas displayed opposite expression pattern in anti miR-335 injection samples. (B) In the late time of cerebral ischemia (24 hrs after eMCAo), the expression of *Hif-1α* and its downstream genes significantly decreased in miR-335 mimic injection samples (*p value* < 0.01) whereas administration of anti miR-335 significantly increased the *Hif-1α*, *Angpt2*, *Mmp9*, *Pai1* and *Vegfa* expression. HIF-1α protein expression was measured by Western blot and displayed the similar expression pattern as *Hif-1α* mRNA. Relative expression (2^-ΔΔCt^) was calculated based on vehicle controls as calibrator and 18S as endogenous control. *p value < 0.05; **p value < 0.01. Each gene was measured 2 times by realtime PCR in triplicates.

During the early time of ischemia when administration of miR-335 mimic was found to be beneficial, HIF-1α protein levels were found to be low. This resulted in a reduction of the expression of *Angpt2*, *Bnip3*, *Mmp9*, *Pai1* and *Vegfa* genes (Fig 5A). On the contrary, during the late time of ischemia where administration of anti miR-335 was found to be beneficial, upregulation of HIF-1α protein was seen and it subsequently increased the expression level of its downstream genes (Fig 5B). Since *Angpt2*, *Bnip3*, *Mmp9*, *Pai1* and *Vegfa* genes are also important key players in cerebral ischemia, it is possible that the overall changes in their expression synergistically contribute in bringing about the beneficial effect of miR-335 in reducing the infarct volume.

## Discussion

### MiR-335 directly targets *Hif-1α*

As the master transcription factor of hypoxia, *HIF-1α* has been widely studied in different diseases and regarded as one of the major candidate for gene therapy in conditions involving hypoxia related pathology. Although several miRNAs have been reported to target *HIF-1α* [16–23], these were mostly done in cancer cells and hence the interaction of miRNAs and *Hif-1α* in cerebral ischemia is still poorly understood. In this study, we aimed to elucidate the interaction between miRNA and *Hif-1α* in cerebral ischemia. A group of miRNAs that are predicted to target *Hif-1a* were shortlisted from bioinformatics databases. Based on the *in silico* prediction, we identified miR-17, -20a, -135a, -203, -335, -338-3p and -93 to be predicted by at least two databases and to be conserved in both rodents and human. These miRNAs were also highly expressed in rat brain and significantly *(p<0*.*05)* dysregulated upon cerebral ischemia. Among these miRNAs, miR-17 and miR-20a have been reported to directly regulate *HIF-1α* in lung cancer cells [16]. Among the other novel predictions, (miR-135a, -203, -335, -338-3p and -93), miR-335 that has two binding sites on the 3'UTR of *Hif-1a* was found to be the most promising regulator of *Hif-1a* expression (Fig 2). Furthermore, Pulkkinen *et al* [37] also observed that miR-335 expression reduced when hypoxic condition was mediated by lack of O_2_ in HUVEC cell line. This suggested that miR-335 is a hypoxia-regulated miRNA. To understand the interaction of miR-335 and *Hif-1α* in *in vitro* ischemic model, we have demonstrated that modulation of miR-335 expression could affect cell viability in an *in vitro* ischemic condition and this occurs *via* regulation of *Hif-1a* (Fig 3). Overexpression of miR-335 in primary neuronal cells subjected to OGD significantly reduced *Hif-1a* expression and subsequently improved cell viability (Fig 3B–3D). Our findings are in agreement with a previous report which demonstrated that administration of *Hif-1a* siRNA could increase neuronal cells viability during OGD [14]. Conversely, Vangeison *et al* [38] has reported that selective loss of HIF-1α function in neurons could increase neuronal susceptibility to hypoxia-induced death which suggested the neuroprotective effect of HIF-1α in neuronal cells. Increasing its intricacy as the essential regulator of hypoxia, HIF-1α was reported to have dual functions in cell death and survival depending on the affected cells types and the type of injury [39,40].

### MiR-335 regulates *Hif-1α* in cerebral ischemia in a biphasic manner

Although *Hif-1α* has been implicated as an important player in cerebral ischemia [14], its function in cerebral ischemic condition is still unclear. miRNAs as gene regulators have demonstrated crucial roles in ischemic stroke pathogenesis [1,25]. Nonetheless, how miRNAs regulate *Hif-1α* upon the onset of the ischemic cascade is still elusive. In our study, we found that HIF-1a expression increased from ischemic stroke onset and demonstrated a biphasic expression pattern as reported by Yeh *et al* [14] and Baranova *et al* [15]. Subsequently, we found that administration of miR-335 mimic immediately after eMCAo inhibited *Hif-1α* expression and consequently reduced the infarct volume by 60% in ischemic stroke models (Fig 4B). Furthermore, it was also found that administration of anti miR-335 at 24 hrs post-occlusion could reduce the infarct volume by 40% (Fig 4D). This result is in concordance with our observation on the biphasic regulation of *Hif-1α* in cerebral ischemia from 0 to 168 hrs. While the decrease in *Hif-1α* expression level is favored for the reduction in infarct volume in the early time of cerebral ischemia (< 24 hrs), an increased expression of *Hif-1α* proved to be beneficial in the late time (> 24 hrs) of permanent middle cerebral artery occlusion model.

### MiR-335 regulates *Hif-1α* downstream gene expression indirectly

**Fig 6:**
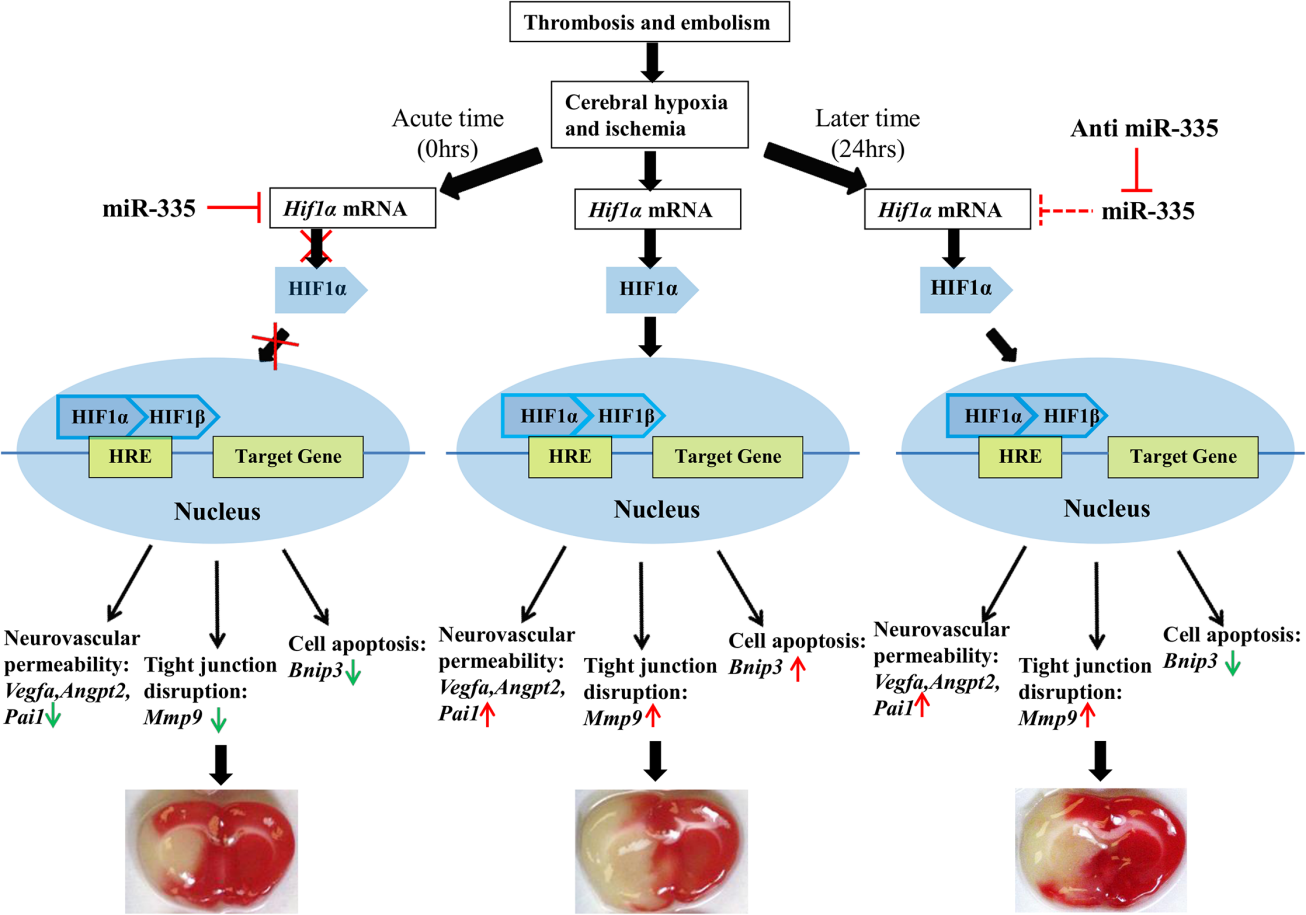
Mechanism of the reduction of infarct volume by miR-335 mimic in the early time and by anti miR-335 in the late time of cerebral ischemia. miR-335 mimic and anti miR-335 could regulate downstream genes of *Hif-1α*, such as *Angpt2*, *Bnip3*, *Mmp9*, *Pai1* and *Vegfa* by modulating *Hif-1α* to result in beneficial outcome in the early (0 hrs) and late time point (24 hrs) of cerebral ischemia respectively. The middle panel of the figure presents that cerebral ischemia activated *Hif-1α* and influenced its downstream genes which brought about a large infarct with severe brain edema. The left panel demonstrates that miR-335 mimic inhibited *Hif-1α* mRNA expression and hence reversed *Hif-1α* downstream genes which resulted in reduced infarct size and edema in the early time of ischemic stroke. The right panel shows that anti miR-335 inhibits miR-335 and therefore releases the respective inhibition on *Hif-1α*, which then activate its downstream protective genes in the late time of cerebral ischemia and subsequently leading to reduced infarct injury and infarct volume.

Benita et al [6] has reported that 81 genes have the potential of being regulated by HIF-1α upon hypoxia in multiple cell types (S1 Table). Based on current literature search, we have added 15 more genes known to be targeted by HIF-1α to the list (S1 Table). Furthermore, to identify the genes that are controlled by HIF-1α in a cerebral ischemic condition, we have performed a DNA microarray experiment on the mRNAs extracted from brain samples of rats subjected to eMCAo. We noticed 56 genes (out of the total 96 listed genes), to be responding to cerebral ischemia with reasonable certainty (S1 and S2 Tables). Nine among these 56 genes have already been documented to be regulated by HIF-1α in cerebral ischemic conditions [14,15,35]. Of these 9 genes, we selected 5 (*VEGFA*, *ANGPT2*, *PAI-1*, *BNIP3*, *MMP9*), as they are known to be involved in crucial pathological processes of cerebral ischemia, such as brain edema, cerebral vascular permeability (*VEGFA*, *ANGPT2*, *PAI-1*), apoptosis (*BNIP3*) as well as blood brain barrier disruption (*MMP9*) [15,35,36]. Changes in *Hif-1a* expression by the administration of either anti miR-335 or miR-335 mimic in cerebral ischemia were accompanied with the corresponding alteration of the downstream genes *Angpt2*, *Bnip3*, *Mmp9*, *Pai1* and *Vegfa* (Fig 5A and 5B). Since none of the genes has a binding site for miR-335 at their respective 3'UTRs, the changes in expression of these genes are most likely an indirect effect attributed by the regulation of *Hif-1a via* miR-335. Being a transcription factor, HIF-1α could regulate these genes by binding to their promoters to increase transcription [41–45]. The changes in expression of these genes could contribute to the reduction in infarct volume (Fig 6) in ischemic stroke. *Angpt2* and *Vegfa* were found to increase cerebrovascular permeability in the early phase of ischemic stroke [46], which is one of the most important factors for the development of brain edema [47] and finally brings about cell death. *Bnip3* is a well-known proapoptotic gene which contributes to apoptosis [15] upon cerebral ischemia. *Mmp9* has been reported to be detrimental in acute stroke by aggravating blood brain barrier (BBB) disruption [48,49], which could lead to intracranial hemorrhage of ischemic stroke. *Pai1* as a plasminogen activator inhibitor was found to deteriorate brain injury by targeting t-PA to delay reperfusion [50] and it was also found to increase neurovascular permeability [51].

### HIF-lα and cerebral ischemia

Several studies have reported that inhibition of *Hif-1α* in acute phase could bring about beneficial effect during cerebral ischemia. For instance, using the neuron-specific *Hif-1a* knock-out mice, Helton *et al* [52] found that *Hif-1a* deficient mice demonstrated reduced infarct volume that is followed by concurrent reduction in the expression of pro-apoptotic genes in 75 min bilateral common carotid artery occlusion. Similarly, Chen *et al* [53] showed that *Hif-1α* specific siRNA inhibited *Hif-1α* and subsequently its downstream apoptosis pathway, to bring about the reduction of infarct volume in acute phase of transient cerebral ischemia. Both, 2-methoxyestradiol (2-ME), a HIF-1α protein inhibitor [54] and hyperbaric oxygen [55] treatment could also inhibit HIF-1α in the acute phase of ischemic stroke to bring about the beneficial effects *via* inhibition of apoptotic genes. Thus, downregulating *Hif-1α* expression through miR-335 mimics, in the early time of cerebral ischemia could prove to be useful in reducing infarct volume of the eMCAo model.

However, although inhibition of *Hif-1α* in the late time (12 hrs post-occlusion) did not show beneficial effect in cerebral ischemia [14], there is no report on the effect of activation of *Hif-1α* in the late time of cerebral ischemia. In this study, we first reported upregulation of *Hif-1α* by administration of anti miR-335 in the late time of cerebral ischemia and displayed that induction of anti miR-335 at 24 hrs post-occlusion could upregulate *Hif-1α* expression and bring about reduction of infarct volume (Fig 4D). The upregulation of *Hif-1α* was accompanied with the changes of its downstream genes (Fig 5B). Some of the downstream genes have been reported to be protective in recovery phase (late phase) of cerebral ischemia and hence maybe involved in the infarct reduction. *Angpt2* and *Vegfa* played an important role in improving angiogenesis and neurogenesis which was favorable for ischemic stroke recovery [56]. *MMP9* as a matrix metalloproteinase could be involved in neuroblast cells migration and hence helped neurogenesis in stroke recovery [57,58]. *PAI1* was found to be anti-apoptotic in the late time of brain injury [59].

### Isoforms of HIF-α

Hypoxia-inducible transcription factors (HIFs), are heterodimeric proteins that are composed of an α and a β subunit. In normoxic conditions, the α subunit is hydroxylated and subsequently undergoes proteasomal degradation via the prolyl-4-hydroxylase domain (PHD) proteins. However, under hypoxic–ischemic conditions, where the oxygen is limited and the PHDs become less active, resulting in the hypohydroxylated HIF-α to be stabilized and induce the transcription of HIF target genes [60].

Three isoforms of HIF-α are known to date; HIF-1α, HIF-2α and HIF-3α. Both the HIF-1α and HIF-2α isoforms have similar domain structures, contains 2 oxygen-dependent degradation domain and are closely related, and both activate HRE-dependent gene transcription [61]. HIF-3α is unique among the HIF-α isoforms, contains only 1 oxygen-dependent degradation domain and its gene is subject to extensive alternative splicing [62].

To date, HIF-1α is the most studied isoform of HIF-α in terms of function and the expression pattern in tissues. However, currently, interest on the functional roles played by HIF-2α and HIF-3α during hypoxia has become evident. Notably, the expression of both HIF-1α and HIF-2α was upregulated under normoxic condition in the forebrain of the homozygous neuron-restricted *Phd2* knockout mice (*nPhd2* ^*Δ/Δ*^). However, under hypoxic conditions, the expression of the HIF-1α was found to be highly expressed compared to HIF-2α in this knockout mouse [63]. Subsequent increase in the mRNA involved in the glycolytic pathway, as well as *Vegf* was noted. The reduction in both the infarct volume and the neuronal cell death/apoptosis was also documented in the acute phase of stroke for this knockout mouse model. Interestingly, the HIF-1α protein in both the wildtype and the *Phd2* knockout mouse brain was similar 24 hrs after ischemia. Using the HIF-1α deficient cell line, 786-O, Hu *et al* [64] demonstrated that HIF-1α distinctly regulates the expression of mRNA encoding enzymes in the glycolytic pathway while HIF-2α promotes tumour growth/progression, a distinctly different function. Pasenan *et al* [65] showed that human HIF-3α variants are induced by hypoxia and also regulated by the changes in the levels of HIF-1α and not by HIF-2α.

Though the co-ordinated regulation of all the 3 isoforms of HIF-α maybe contributing to the physiological changes during hypoxia, it appears that it is the activity of HIF-1α isoform possibly conferring neuroprotection or beneficial effects (to the cells) during cerebral ischemia. The HIF-1α maybe executing its effect *via* the glycolytic and cell survival pathways. Therefore, regulating the expression of HIF-1α by using its endogenous regulator, miR-335 could prove beneficial in stroke outcome.

## Conclusions

In this study, we have established that miR-335 can directly modulate *Hif-1α* gene expression in both *in vitro* and *in vivo* cerebral ischemic conditions. In *in vivo* study, we have also observed that administration of miR-335 mimic could bring about a decrease in expression of *Hif-1α* and consequently a reduction in infarct volume in the early time of cerebral ischemia whereas administration of anti miR-335 could reduce infarct volume in the late time of cerebral ischemia. By regulating *Hif-1α* expression, miR-335 also indirectly affects the expression of the downstream genes, which are crucial in the maintenance of cell viability and blood brain barrier integrity.

## Supporting Information

S1 Table List of 96 genes regulated by HIF-1α. Eighty one genes that were predicted to be regulated by HIF-1α by Benita et al [6] and fifteen more genes (shown in bold), reported by several other workers (see manuscript for corresponding references) as HIF-1α downstream genes involved in cerebral ischemia are listed. 56 of these genes (indicated with ticks) have been found to be differentially expressed in our DNA microarray experimental data (GEO No: GSE46267) from eMCAo models.
(XLS)

S2 Table List of 56 genes identified to be regulated by HIF-1α under cerebral ischemic condition (DNA array data; GSE46267). The expression of these 56 genes is presented as fold change.
(XLS)
